# High‐Performance Stretchable Thermoelectric Generator for Self‐Powered Wearable Electronics

**DOI:** 10.1002/advs.202206397

**Published:** 2023-02-17

**Authors:** Wusheng Fan, Zijian An, Feng Liu, Ziheng Gao, Min Zhang, Chenguang Fu, Tiejun Zhu, Qingjun Liu, Xinbing Zhao

**Affiliations:** ^1^ State Key Laboratory of Silicon Materials and School of Materials Science and Engineering Zhejiang University Hangzhou 310027 China; ^2^ Biosensor National Special Laboratory Key Laboratory for Biomedical Engineering of Education Ministry Department of Biomedical Engineering Zhejiang University Hangzhou 310027 China; ^3^ Shanxi‐Zheda Institute of Advanced Materials and Chemical Engineering Taiyuan 030000 China

**Keywords:** self‐powered, thermoelectric generator, thermoelectric performance, wearable electronics

## Abstract

Wearable thermoelectric generators (TEGs), which can convert human body heat to electricity, provide a promising solution for self‐powered wearable electronics. However, their power densities still need to be improved aiming at broad practical applications. Here, a stretchable TEG that achieves comfortable wearability and outstanding output performance simultaneously is reported. When worn on the forehead at an ambient temperature of 15 °C, the stretchable TEG exhibits excellent power densities with a maximum value of 13.8 µW cm^−2^ under the breezeless condition, and even as high as 71.8 µW cm^−2^ at an air speed of 2 m s^−1^, being one of the highest values for wearable TEGs. Furthermore, this study demonstrates that this stretchable TEG can effectively power a commercial light‐emitting diode and stably drive an electrocardiogram module in real‐time without the assistance of any additional power supply. These results highlight the great potential of these stretchable TEGs for power generation applications.

## Graphical abstract

A stretchable thermoelectric generator (TEG) is fabricated, which exhibits both outstanding output performance and comfortable wearability for human body heat harvesting. The stretchable TEGs are enough for driving an electrocardiogram module in real time without the assistance of an additional power supply.

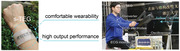

## 1 Introduction

The booming flexible electronics technology has greatly speeded up the development of wearable biomedical monitoring devices.^[^ ^1^, ^2^, ^3^ ^]^ The realization of long‐time and continuous monitoring of daily healthy is possible with self‐powered wearable devices that are capable of operating sustainably, measuring imperceptibly, and wearing comfortably.^[^ ^4^, ^5^, ^6^ ^]^ However, maintaining an uninterrupted power supply is still the bottleneck for wearable electronic technology.^[^ ^7^ ^]^ One promising solution is to develop wearable self‐powered technologies that can collect energy from the human body or environment, i.e., battery‐free wearable electronics.^[^ ^8^ ^]^ The thermal energy that spreads all over the human skin is a stable and reliable energy source for self‐powered devices.^[^ ^9^ ^]^ Thermal energy can be converted into electricity for powering electronic devices by thermoelectric generators (TEGs). This kind of energy conversion, which is not affected by the intensity of electromagnetic waves and is free of body movement or catalysts, can guarantee long‐time usage.^[^ ^10^, ^11^, ^12^ ^]^

In reality, the practical applications of self‐powered wearable electronics with TEGs remain scarce. This is because most wearable electronics require a power input ranging from several microwatts to tens of milliwatts, which is higher than the output performance of the vast majority of existing wearable TEGs.^[^ ^13^, ^14^, ^15^, ^16^, ^17^, ^18^ ^]^ The development of wearable TEGs with higher output performance is essential to promote practical applications of self‐powered wearable devices.^[^ ^16^ ^]^ To improve the performance of wearable TEGs, several aspects, including the usage of good TE materials, the optimization of heterogeneous interfaces, and the topological design of device structure, were commonly employed.^[^ ^9^, ^19^, ^20^, ^21^ ^]^

Bulk Bi_2_Te_3_‐based alloys, the only commercialized TE materials,^[^ ^22^, ^23^, ^24^ ^]^ have generally been employed as TE legs in wearable TEGs,^[^ ^25^, ^26^, ^27^, ^28^, ^29^, ^30^, ^31^, ^32^, ^33^, ^34^, ^35^, ^36^, ^37^, ^38^, ^39^ ^]^ which however are naturally brittle near room temperature. To realize the wearability of electronic devices, the brittle Bi_2_Te_3_‐based TE legs are generally encapsulated in flexible substrates,^[^ ^25^, ^26^, ^27^, ^28^, ^29^ ^]^ such as polydimethylsiloxane (PDMS) and Ecoflex. However, the low thermal conductivity of flexible substrates leads to a significant loss of thermal energy in the substrate, resulting in an unsatisfactory power output of wearable TEGs. To reduce the loss of thermal energy in the substrates, the thermal design of heterogeneous interfaces has recently been performed.^[^ ^16^, ^29^, ^39^, ^40^ ^]^ Lee et al. reported a compliant TEG with magnetically self‐assembled metal particles in a PDMS substrate, achieving reduced thermal energy loss in substrates and enhanced output performance.^[^ ^29^ ^]^ Wang et al. filled the PDMS substrate with thermally conductive filler, i.e., Fe_3_O_4_ nanoparticles decorated on boron nitride (BN@Fe_3_O_4_), enabling the increased thermal conductivity of substrate (*κ*_sub_) and thus, decreasing the thermal energy loss.^[^ ^40^ ^]^ Although these strategies are helpful to reduce the thermal energy loss, the *κ*_sub_ is however still smaller than that of the TE legs and thus considerable heat loss is unavoidable. To further maximize the utilization of thermal energy, a feasible alternative way is to design a self‐supported device structure that can get rid of the low‐thermal‐conductivity substrates. However, high‐performance substrate‐free TEGs have rarely been investigated for wearable electronics. The difficulty lies in how to achieve a substrate‐free structure with guaranteed wearability and stretchability.

Here, we report the realization of substrate‐free stretchable TEGs (s‐TEGs) that can simultaneously exhibit excellent thermal utilization efficiency and comfortable wearability for human body heat harvesting. The design of a substrate‐free structure minimizes thermal energy loss and thus achieves a giant output power density only utilizing human body heat. When worn on the forehead at an ambient temperature of 15 °C, our fabricated s‐TEGs produce a maximum power density of 13.8 µW cm^−2^ in breezeless conditions, which can rapidly increase to 71.8 µW cm^−2^ at an air speed of 2 m s^−1^, generally achieved during the normal walking. In reality, the stretchable TEGs are enough for lighting a commercial light‐emitting diode (LED) only in a breezeless condition and driving an electrocardiogram (ECG) module in real‐time without the assistance of an additional power supply, demonstrating their prospect for wearable electronics.

## 2 Results and Discussions

### 2.1 The Design of s‐TEG

**Figure 1:**
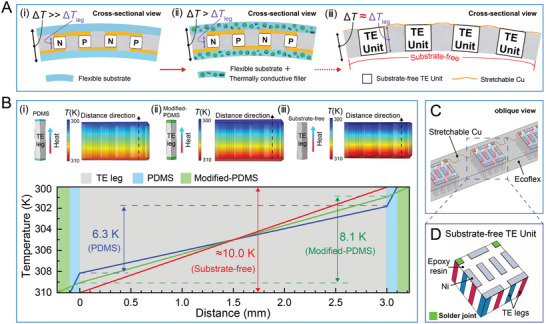
Effect of substrate‐free structure on the performance of TEG. A) Schematic illustration showing three types of wearable TEGs with different substrates, including a flexible substrate (i), a flexible substrate with thermally conductive filler (ii), and a substrate‐free one (iii). B) Finite element analysis (FEA) results show the temperature distribution on the cross section of the TEGs with structures of PDMS substrate (i), PDMS/BN@Fe_3_O_4_ substrate (ii), and substrate‐free (iii) for a given temperature difference of 10 K. C) The structure of the substrate‐free s‐TEG with TE units connected by stretchable Cu electrodes and embedded in Ecoflex. D) Schematic diagram of TE units, connected mutually by stretchable copper wires.

A large temperature difference (Δ*T*_leg_) across the TEG is critical to achieving a high maximum output power *P*_max_, which is expressed as *P*_max_ = $\frac{N^{2}{\left(S_{P}-S_{N}\right)}^{2}}{4R}\Delta T_{\mathrm{leg}}^{2}$,^[^ ^19^ ^]^ where *N*, *S*_P_, *S*_N_, and *R* are the number of p‐n couples, the Seebeck coefficients of the p‐ and n‐type TE legs, and the resistance of the device, respectively. For the application of wearable electronics, although the Δ*T*_leg_ between the human skin and room temperature can be larger than 10 K, the effective Δ*T*_leg_ across the TE legs is usually only about 1–2 K, owing to the unavoidable heat loss in the interfaces.^[^ ^9^ ^]^ To effectively establish a large Δ*T*_leg_, we proposed the design of the substrate‐free s‐TEG, consisting of miniature TE units that are connected by stretchable electrodes and polymers. **Figure** **1**A schematically illustrates the structure (cross‐sectional view) of our substrate‐free s‐TEG (iii) in comparison to the previously reported wearable TEGs (i and ii). Flexible materials, such as PDMS, have generally been employed as substrates in previous studies, but their high thermal resistance significantly impedes heat transfer from the heat source to the TE legs, resulting in the much smaller effective Δ*T*_leg_, if compared to the total temperature difference across the TEG system (Δ*T*). Filling the flexible substrate with thermally conductive filler, e.g., PDMS/BN@Fe_3_O_4_,^[^ ^40^ ^]^ was recently employed to improve *κ*_sub_, which is helpful to enhance the heat transfer to TE legs. To further suppress the heat loss, a substrate‐free s‐TEG integrated with miniaturized TE units and stretchable electrodes could be an effective approach to maximize the Δ*T*_leg_.

The effect of substrate‐free on the Δ*T*_leg_ built across the TEG is theoretically studied via finite element analysis (FEA). The FEA results (Figure 1B) show the temperature distribution on a cross‐section of the three types of TEGs by setting the total Δ*T* between the top (300 K in an ambient environment) and bottom boundaries (310 K) as 10 K, which is generally achievable between the ambient environment and human skin. The details of the modeling can be found in Figure S1 and Table S1 (Supporting Information). In the cases of the TEG with substrates, although the thickness of the PDMS (i) and modified‐PDMS (ii, i.e., PDMS/BN@Fe_3_O_4_) supporting layers (120 and 220 µm, respectively), are much thinner than the height of the TE leg (3‐mm thickness), the Δ*T*_leg_ (6.3 and 8.1 K, respectively) is still significantly lower than the given Δ*T* (10 K), manifesting a significant loss of thermal energy across the flexible substrates. In contrast, s‐TEG with a substrate‐free structure can maximize the utilization of Δ*T*, showing a Δ*T*_leg_ of nearly 10.0 K. Figure 1C schematically illustrates the designed structure of the s‐TEG with TE units connected by stretchable Cu electrodes and embedded in stretchable Ecoflex. Obviously, substrate‐free TE units are essential for the formation of s‐TEG, which is hopeful to establish a large Δ*T*_leg_ and thus realize high output performance. In addition, this structure requires that each unit connects to two adjacent units by only two solder joints on one side (Figure 1D). It means that our s‐TEG is less likely to break (usually occurs at the solder joint) during physical deformation (e.g., stretching) than typical TEG with much more solder joints (i.e., two per TE leg).

### 2.2 Fabrication of TE Unit

**Figure 2:**
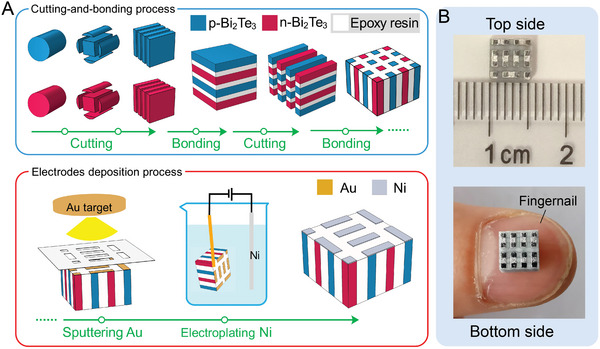
The fabrication process of TE unit. A) Schematic illustration of the assembly procedure of the TE unit, including the cutting‐and‐bonding process and electrode deposition process. B) Photographs of the fabricated 5.5 × 5.5 mm^2^ TE unit, which is smaller than a fingernail.

The preparation of a substrate‐free TE unit is the basis for realizing designed s‐TEG. The assembly process of the TE unit is illustrated schematically in **Figure** **2**A. In brief, high‐performance Bi_2_Te_3_‐based TE materials were repeatedly cut and bonded to obtain a 3D n‐p array.^[^ ^33^, ^41^ ^]^ The Au layers were subsequently deposited by a radio frequency (RF) magnetron sputter to join the p‐ and n‐pairs in a series circuit. Finally, the TE unit was achieved after the electrochemical deposition of Ni on the Au layers to reduce the resistance of the electrodes. The optical photographs of our fabricated TE unit (Figure 2B) show that 8‐pair TE legs (0.6 × 0.6 mm^2^ in cross section and 3 mm in height, with a gap of 0.8 mm between adjacent legs) are evenly distributed in an area of 5.5 × 5.5 mm^2^, which is even smaller than a fingernail.

### 2.3 Stability and Output Performance of TE Unit

**Figure 3:**
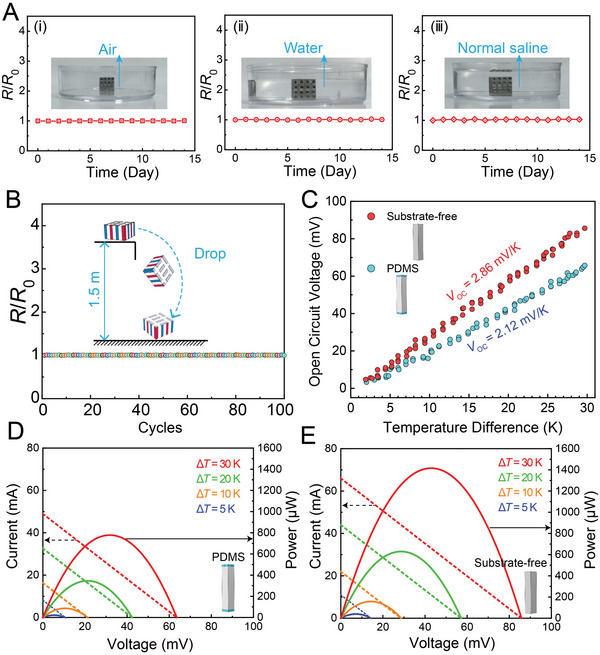
Stability and output performance of TE unit. A) Relative resistance changes over 14 d under different environments, including air environment (i), water environment (ii), and normal saline environment (iii). B) Relative resistance changes over 100 dropping cycles with a height of 1.5 m. C) Comparison of experimental measured open‐circuit voltage of TE unit with structures of PDMS substrate and substrate‐free. The experimentally measured output performance of TE units with structures of D) PDMS substrate and E) substrate‐free showing current and power as a function of voltage.

The fabricated TE units showed high stability and excellent output performance, which are suitable as basic elements for the assembly of s‐TEG. **Figure** **3**A demonstrates the performance stability of the TE unit by monitoring the change in relative resistance (*R*/*R*_0_, where *R* is the measured resistance after treatment and *R*_0_ represents the initial resistance) under different environments, i.e., ambient air (i), pure water (ii), and normal saline (0.9 wt% NaCl, iii). The relative resistance remains almost unchanged when the TE units were exposed to these environments for 14 d, indicating the high stability of the TE unit in human body heat harvesting even under sweating conditions. Moreover, the TE unit was repeatedly dropped up to 100 cycles from a height of 1.5 m. Despite the repeated dropping cycles, the TE unit exhibits a strong impact resistance capacity with almost unchanged *R*/*R*_0_, as shown in Figure 3B. These results demonstrate that our TE unit has the advantage of high stability in wearable applications.

To examine the effect of our substrate‐free structure on power generation, we investigated the output performance of the 8‐pair TE unit by experimental measurements. As a comparison, a control device coated with a thin PDMS substrate (120‐µm‐thick), which is widely used as a substrate in wearable TEGs, was also fabricated and measured. Measurement details are presented in the Experimental Section. The advantages of the substrate‐free structure in terms of output characteristics are presented in Figure 3C–E. The TE unit generates an open‐circuit voltage (*V*_OC_) of 86 mV at Δ*T* of 30 K, which is 35% higher compared to the control device (Figure 3C). As a result, Figure 3D,E shows that the TE unit provides 182% higher output power (1416 µW) in comparison with that of the control device with PDMS substrate (778 µW) at a Δ*T* of 30 K. The comparison in the output performance of the state‐of‐the‐art TEGs with various substrates (i.e., substrate‐free, low *κ*_sub_ of PDMS, polyimide, silicone composite, high *κ*_sub_ of Ag—Ni particles in PDMS, Ecoflex/AlN, and ceramic sheets) is summarized in Table S2 (Supporting Information).^[29,34,35,37–39]^ Among them, the substrate‐free TE unit shows the highest normalized output voltage per unit area (9.45 mV K^−1^ cm^−2^), which is even comparable to the commercial TE devices despite having a high *κ*_sub_ of ceramic sheets.^[^ ^38^ ^]^ Furthermore, our TE unit shows an outstanding performance in the normalized power density, i.e., 5.13 µW cm^−2^ K^−2^, one of the highest values for TEGs reported so far. These results highlight that our substrate‐free structure is an effective approach to greatly improving the output performance of TEGs.

### 2.4 Lego‐Like Assembly, Mechanical Reliability, and Wearability of s‐TEGs

**Figure 4:**
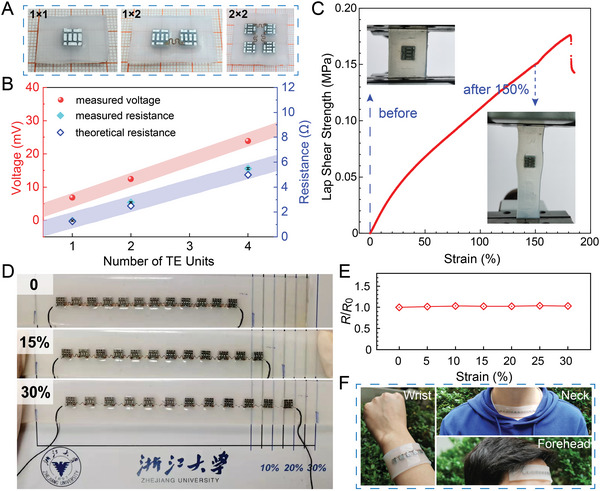
Lego‐like assembly, mechanical reliability, and wearability of s‐TEGs. A) Photographs of substrate‐free s‐TEGs with various TE unit arrays (i.e., 1 × 1, 1 × 2, and 2 × 2). B) Dependences of open‐circuit voltage and resistance on the number of TE units. C) Lap shear test showing structural stability between Ecoflex and epoxy resin under uniaxial stretching. The inset photographs show the sample under a strain of 0 and 150%. D) Photographs show the strip‐shaped s‐TEG comprising 12‐TE units under a uniaxial strain of 0, 15%, and 30%. E) Relative resistance changes as a function of uniaxial strain from 0 to 30%. F) Photographs of strip‐shaped s‐TEGs attached conformably to various human skin locations (wrist, neck, and forehead).

Due to the small area and excellent output performance of the TE units, high‐performance wearable s‐TEGs are expected to be realized by combining multiple TE units. Here, we fabricated substrate‐free s‐TEGs with TE units connected by stretchable Cu electrodes and embedded in stretchable Ecoflex. Fabrication details are given in the Experimental Section and Figure S2 (Supporting Information). The optical photographs of s‐TEGs with various TE units arrays (i.e., 1 × 1, 1 × 2, and 2 × 2) are presented in **Figure** **4**A, showing the Lego‐like construction of our s‐TEGs. To investigate the output performance of these s‐TEGs with different arrays, a series of measurements of the *V*_OC_ and *R*_0_ were conducted, as shown in Figure 4B. The experimental setup for performance evaluation of the s‐TEGs worn on a subject's wrist can be found in Figure S3 (Supporting Information). Both *V*_OC_ and *R*_0_ showed a linear increase with the increased number of TE units, indicating that the Lego‐like assembly process is effective with negligible performance degradation. Noted that the Lego‐like construction allows users to customize TEGs using TE units in series or parallel to achieve targeted form factor, construction, output voltage, and power based on specific thermal conditions and output of TE units.^[^ ^39^ ^]^

The adhesion between the epoxy resin of TE units and Ecoflex enables the construction of s‐TEG by interconnecting the individual TE units. To investigate the adhesion strength at the contact surface between the epoxy resin and Ecoflex, we measured lap shear strength as a function of strain during uniaxial stretching as shown in Figure 4C. It can be observed that the strength value reaches 0.176 MPa with strain over 170%, showing high structural stability under stretching. To further investigate the performance in practical applications, we prepared a strip‐shaped s‐TEG with 12‐TE units connected in series in a row and distributed evenly in an area of about 11.5 × 0.6 cm^2^ (see Figure S4, Supporting Information), whose shape is well suited for wearable applications. Thanks to the small area of the TE units and the stretchability of the Ecoflex and Cu interconnects, the strip‐shaped s‐TEG comprising 12‐TE units shows stretchability up to as high as 30% as presented in Figure 4D. The *R*_0_ of the s‐TEG is 16.0 Ω (see Figure S5, Supporting Information) and remains constant during the stretching process (Figure 4E). Therefore, our s‐TEGs can be conformably attached to various parts of the human body (wrist, neck, and forehead) as shown in Figure 4F, demonstrating the comfortable wearability of s‐TEGs for human body heat harvesting.

### 2.5 The Output Performance of s‐TEG Utilizing Human Body Heat

**Figure 5:**
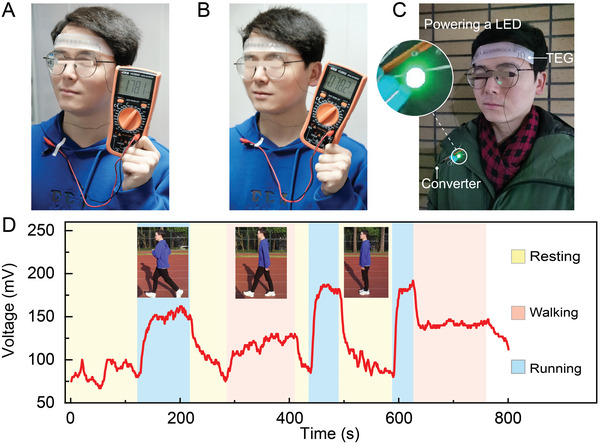
The output performance of s‐TEG utilizing human body heat. Photographs of the measured *V*_OC_ of the s‐TEG attached to the forehead at an air speed of A) 2 m s^−1^ and B) 0, respectively. C) Photograph of LED powered by the s‐TEG in a breezeless condition. D) Daily performance of the s‐TEG worn on the forehead at different activities, i.e., resting, walking, and running.

As mentioned above, the s‐TEG with a creative substrate‐free structure is promising to achieve excellent output performance. We then designed a series of human body heat harvesting experiments to evaluate the performance of the s‐TEG. The ambient temperature for all experiments in this work was around 15 °C. As shown in **Figure** **5**A,B, the *V*_OC_ of the s‐TEG was measured by attaching it to the forehead at two different test conditions, i.e., one with the air speeds of 2 m s^−1^ (movement state) and the other of zero (static state). The s‐TEG generates a *V*_OC_ of 178.1 mV and a power density of 71.8 µW cm^−2^ at an air speed of 2 m s^−1^. Even under the breezeless condition, the *V*_OC_ and power density of the s‐TEG can reach decent values of 78.2 mV and 13.8 µW cm^−2^, respectively. One can note that these outstanding output performances are among the highest reported values for wearable TEGs on human skin (see Table S3, Supporting Information). Thanks to the outstanding output performance, our s‐TEG can power a commercial LED via a boost converter (LT3108) at a breezeless condition (Figure 5C and Video S1, Supporting Information).

Figure 5D further shows the daily performance of the fabricated s‐TEG worn on a subject's forehead, which was tested in an outdoor stadium for ≈15 min. The subject was asked to perform resting, walking, and running while the *V*_OC_ of the s‐TEG was automatically measured using data recording equipment (sample rate: 1 Hz). The daily performance of s‐TEG is highly correlated with the activity status of the subject, due to the variation in cooling efficiency of the cold side at different airflow velocities. In general, the s‐TEG generates output voltages exceeding 75 mV at rest, about 120–140 mV while walking, and over 150 mV while running. The maximum output power of the s‐TEG is calculated to be 625 µW, which is high enough to power some wearable medical monitoring devices, as shown next.

### 2.6 Demonstration of Self‐Powered ECG System

**Figure 6:**
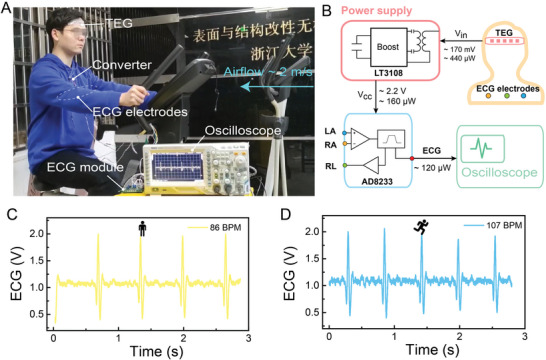
Demonstration of self‐powered ECG system using s‐TEG. A) Photograph of the experimental setup used to drive the ECG module utilizing human body heat. B) Functional block diagram of the self‐powered ECG operating system. ECG signal displayed at C) rest and D) after cycling.

To demonstrate the versatile applications of the high power generation capacity of our s‐TEG, **Figure** **6**A–E shows that our strip‐shaped s‐TEG can stably drive an ECG module in real‐time without the assistance of any additional power supply, which is distinct from the previously reported TEG‐powered ECG with the assistance of the charge capacitor and a polymer‐based flexible heat sink.^[^ ^42^ ^]^ Figure 6A shows a photograph of the experimental setup that was used to drive the ECG module utilizing human body heat. The subject was asked to simulate cycling on exercise equipment, and the air velocity of 2 m s^−1^ was generated by a fan to simulate the airflow effect of outdoor jogging. The s‐TEG was attached to the subject's forehead to generate electricity, which drove the ECG module via a boost converter. The ECG signal was first collected by ECG electrodes placed on the chest, then processed in the ECG module, and finally displayed and recorded by an oscilloscope.

The functional block diagram is shown in Figure 6B, and a detailed circuit diagram can be found in Figure S6 (Supporting Information). The power management module consists of the LT3108 ultralow‐voltage boost converter and some passive devices. It is responsible for boosting the millivolt level TEG output voltage to about 2.2 V to power the regular electronics and storing the additional energy in the capacitor. The ECG module, which consists of an AD8233 amplifier and some resistors and capacitors, provides a complete ECG analog front end and consumes only about 50 µA. Although the s‐TEG generated about 440 µW of power in this case, there is a large drop in the power supply after the boost process due to the imperfectly matched impedance. The resulting power (nearly 160 µW) drove an ECG module and enabled continuous monitoring of cardiac load. As illustrated in Figure 6C,D, the ECG waveforms show how the subject's heart rate varied, from 86 BPM (beat per minute) at rest to 107 BPM after cycling, with a Video S2 (Supporting Information) showing the test scenario and procedure. The result validates the possibility of human body heat as an energy source for continuous sign monitoring devices, which is an exciting advance for wearable continuous sign monitoring to achieve unlimited endurance. It is conceivable that an uninterrupted power supply for wearable biomedical monitoring devices will be successfully achieved to meet the need for long‐term and continuous monitoring of daily health with the development of self‐powered wearable TEGs.

## 3 Conclusion

A strip‐shaped s‐TEG with a substrate‐free structure has been fabricated, which exhibits both outstanding output performance and comfortable wearability for human body heat harvesting. This structure is realized by integrating the high‐performance TE units, stretchable Cu electrodes, and Ecoflex through a Lego‐like assembly process. The resulting s‐TEG exhibits comfortable wearability on the human body and maximizes utilization of thermal energy to achieve record‐high power density, which worn on the forehead produces a maximum power density of 13.8 µW cm^−2^ at a breezeless condition, and even as high as 71.8 µW cm^−2^ at an air speed of 2 m s^−1^. We demonstrate that our s‐TEG generates enough high electricity from the human body to power a commercial LED under breezeless conditions. The outdoor test shows a high power of 625 µW reached in daily activities by our s‐TEG. Importantly, we further demonstrate that the outstanding output power can stably drive an ECG module in real‐time without the assistance of any additional power supply, which is an exciting advance for wearable continuous sign monitoring utilizing human body heat. This work opens up a new design solution for realizing high‐performance TE devices, paving the way toward the realization of self‐powered wearable electronics.

## 4 Experimental Section

### Fabrication of TE Unit

Bi_2_Te_3_ bulk TE materials were purchased from Changshan Wangu Electronic Technology Co., Ltd., and the TE properties of p‐ and n‐type Bi_2_Te_3_ materials are shown in Figure S7 (Supporting Information). The electrical conductivity and Seebeck coefficient were measured using a commercial Linseis LSR‐3 system in a helium atmosphere. The thermal conductivity (*κ*) was calculated from the thermal diffusivity (*D*), density (*ρ*), and constant‐pressure specific heat (*C*_p_) using the relationship *κ* = *DρC*_p_, where *D* was measured on a Netzsch LFA 467 apparatus, *ρ* was measured by the Archimedes method, and *C*_p_ was estimated using the Dulong–Petit law.

The TE unit was fabricated by bonding‐and‐cutting and electrode deposition processes. In this work, TE legs with the area of 0.6 × 0.6 mm^2^ and a gap of 0.8 mm between adjacent legs in the TE units were used, as the smaller dimensions were difficult to obtain by the manual preparation methods. The height of the TE legs was set to be 3 mm to ensure good wearability and performance of the TE units. In a typical procedure, the cylindrical p‐ and n‐Bi_2_Te_3_ materials were first cut into slices (0.6‐mm‐thick) by a commercial automatic cutting machine (STX‐202A, Shenyang Kejing), and alternately bonded to each other using an epoxy resin/12 wt% hollow glass bead composite to form a layer‐structured cube. The composite was prepared by directly mixing the epoxy resin (88 wt%, M03, Kunshan Jiulimei) and hollow glass beads (12 wt%, K46, 3 M), which were also used as an insulating medium between each p‐ and n‐sheet. The layer‐structured cube was cut into 0.6‐mm‐thick slices and bonded to each other again to obtain a checkboard‐like sheet.

The electrodes were subsequently deposited to join the p‐ and n‐pairs in a series circuit. Specifically, a mask was laid over the above checkboard‐like sheet and Au electrodes were deposited by a RF magnetron sputter (VTC‐1RF, Hefei Kejing). The sputtering power was set at 40 W, the working pressure was 10 Pa, and the sputtering time was 20 min. Note that before magnetron sputtering, the surfaces of the checkboard‐like sheet were cleaned using plasma cleaner (PDC‐36G, Hefei Kejing) to improve the bonding strength between TE legs and Au electrodes. Finally, the TE unit was achieved after electrochemical deposition of Ni electrodes on Au to reduce the resistance of the electrodes. A CHI660D electrochemical workstation (Shanghai CH Instruments Co.) was employed with a current density setting of 1.5 A dm^−2^ and a deposition time of 1 h.

### Fabrication of Lego‐Like Substrate‐Free s‐TEG

To connect all solid‐state TE units in series to form a stretchable TE device, the stretchable polymer matrix “Ecoflex” (00‐30, Smooth‐On) and stretchable Cu electrodes were used in this work. A certain number of the TE units were first equally spaced at 3.5 mm intervals within a 3 mm high mold. The interval of 3.5 mm ensures good stretchability of the TEGs. The TE units can be arranged in one or more rows in a Lego‐like format. Then, Ecoflex was filled into the gap to bond the TE units together. The mold was then removed after 4 h when the ecoflex was fully cured at room temperature. Finally, the stretchable Cu electrodes were assembled with the TE units by soldering to join the TE units in a series circuit, generating a substrate‐free s‐TEG.

### TEG Output Characterization

The output performance of the TE unit as a function of Δ*T* was tested using a homemade setup (see Figure S8, Supporting Information). Two T‐type ultrathin thermocouples (wire diameter, 0.08 mm) were employed to test the hot‐side and cold‐side temperatures of the TE unit. To accurately measure the surface temperatures, two thin copper sheets (1 mm thickness) with a tiny hole in the center were placed on two sides of the TE unit, and the two thermocouple tips were located in those holes. The steady heat was provided by a flat heater (X150, Xinhaomai Co.). *V*_OC_ and temperature were simultaneously measured per second using a dedicated testing device (Guangzhou Leizig Electric Machinery Co.) and then outputted to a computer. This dedicated testing device was also employed to continuously monitor the output voltage of the s‐TEG under different motion states of the human body. The *R*_0_ and *V*_OC_ of s‐TEGs with different arrays attached to a human wrist were measured using a commercial AC Electrical Load (AT527L, Applent) under steady‐state conditions. For resistance measurements, at least three samples were measured and the average value was used. For a clear presentation, a commercial handheld multimeter (VC890D, Victor) was used to measure the *V*_OC_ of the strip‐shaped s‐TEG comprising 12‐TE units attached to a human forehead.

### Mechanical Characterization

The drop test was carried out by manual work. The TE unit was repeatedly dropped from a height of 1.5 m onto a concrete floor and subsequently, the resistance was repeatedly measured. The adhesion strength at the contact surface between the epoxy resin and Ecoflex was characterized by a lap shear test in a commercial Electronic Universal Testing Machine (5943, Instron) with a speed of 2 mm min^−1^. The stretch test was performed on a homemade setup. First, mark the point corresponding to the stretch rate on the paper, and then stretch the device to the corresponding point. The above‐mentioned AC Electrical Load was employed to measure resistance during the stretching process.

### TEG‐Powered Electrocardiogram Experiment during Exercise

The electronic circuit was designed using Altium Designer PCB design software. The detailed circuit diagram is shown in Figure S6 (Supporting Information). The main components include a power management unit (LT3108, Analog Devices), an ECG amplifier (AD8233, Analog Devices), and passive components. The ECG waveform is displayed and recorded by an oscilloscope (DS2202A, Rigol). The s‐TEG was attached to a subject's forehead. The output voltage was boosted by the power management unit and supplied to the ECG module. The ECG measurement uses a standard three‐electrode configuration with left chest, right chest, and right leg drives. A fan was used to simulate the airflow during movement.

## Conflict of Interest

The authors declare no conflict of interest.

## Supporting information

Supporting Information

Supplemental Video 1

Supplemental Video 2

## Data Availability

The data that support the findings of this study are available from the corresponding author upon reasonable request.
