# Supplementary material for: High‐Performance Stretchable Thermoelectric Generator for Self‐Powered Wearable Electronics
Source: Adv Sci (Weinh). 2023 Feb 17;10(12):2206397. doi: 10.1002/advs.202206397 (PMC10131832; doi:10.1002/advs.202206397)
Supplement: Supplementary file 1 — Supporting Information [file ADVS-10-2206397-s003.pdf]

Supporting Information

High-performance stretchable thermoelectric generator for self-powered wearable electronics

Wusheng Fan,[a] Zijian An,*[b] Feng Liu,[a] Ziheng Gao,[a] Min Zhang,[a] Chenguang Fu,[a] Tiejun Zhu,[a] Qingjun Liu,[b] and Xinbing Zhao[a]*

[a]State Key Laboratory of Silicon Materials, and School of Materials Science and Engineering, Zhejiang University, Hangzhou 310027, China.

[b]Biosensor National Special Laboratory, Key Laboratory for Biomedical Engineering of Education Ministry, Department of Biomedical Engineering, Zhejiang University, Hangzhou 310027, China.

*These two authors contributed equally to this work.

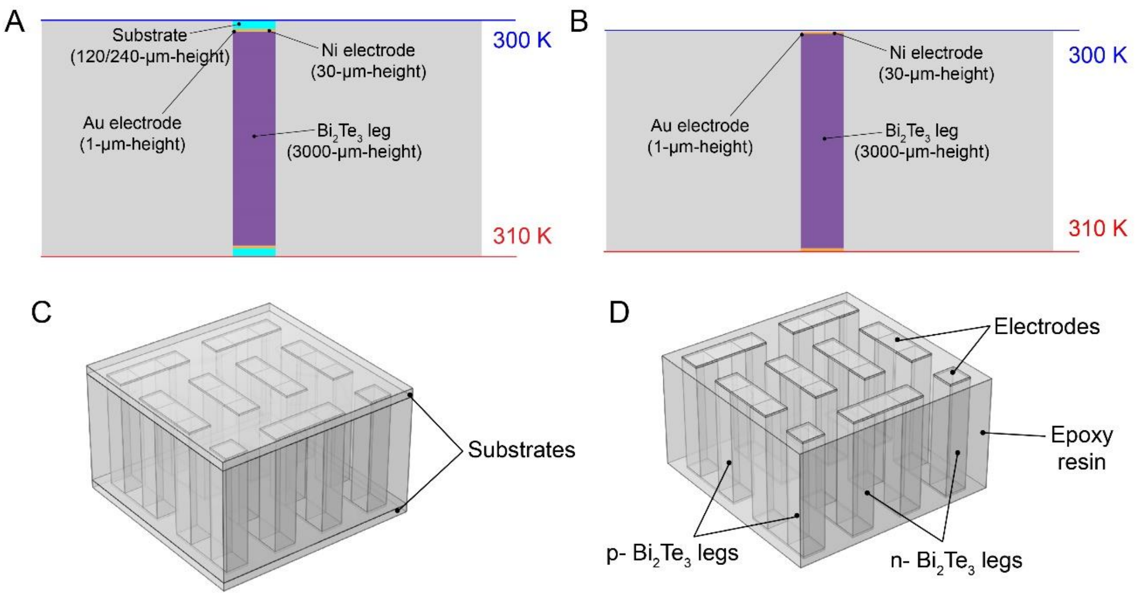

Figure S1 Finite element analysis (FEA) modeling for assessing the performance of TEGs with different substrate structures. (A), (B) Schematic illustration of unit compliant TEG with a single Bi_2Te_3 leg for FEA modeling. The TEGs with different structures of flexible substrates (A) and substrate-free (B) were characterized for a given temperature difference of 10 K. (C), (D) Modelling details to indicate each component of the TEGs with different structures of the flexible substrate (C) and substrate-free (D).

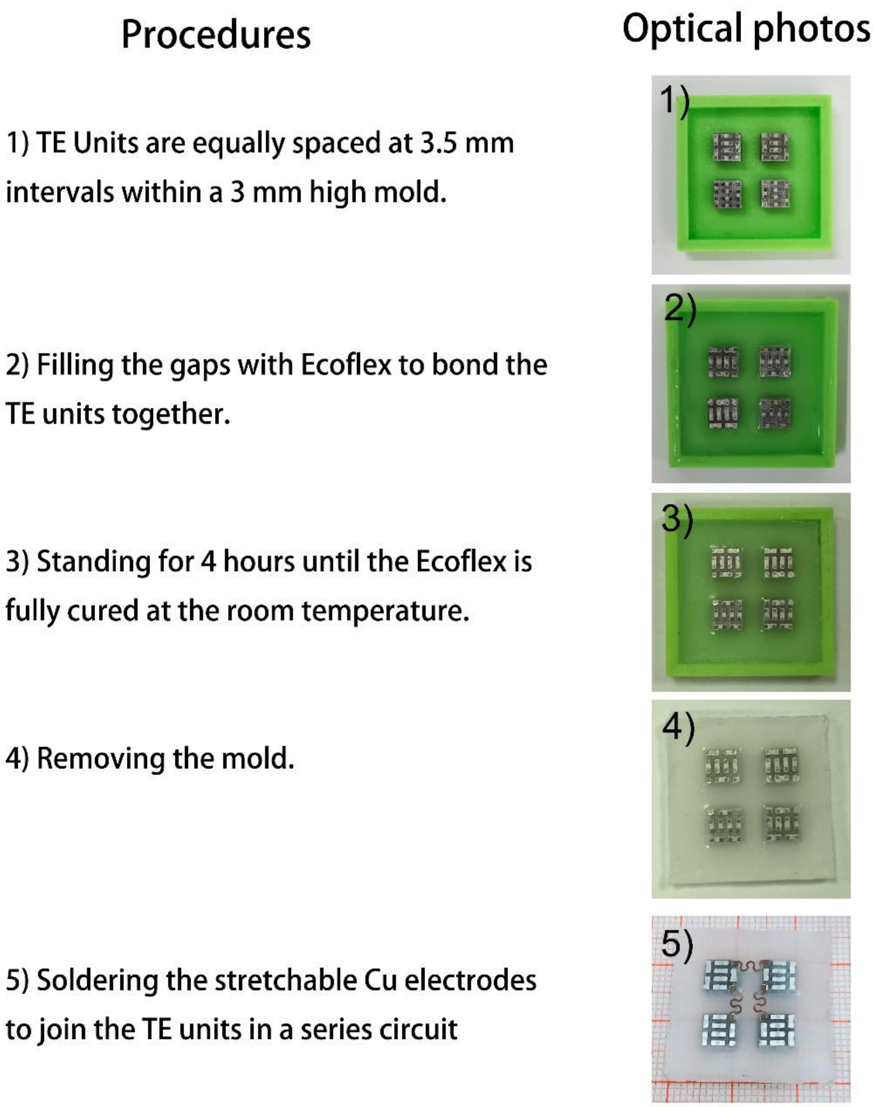

Figure S2 Schematic diagram of step-by-step Lego-like assembly processes of s-TEG.

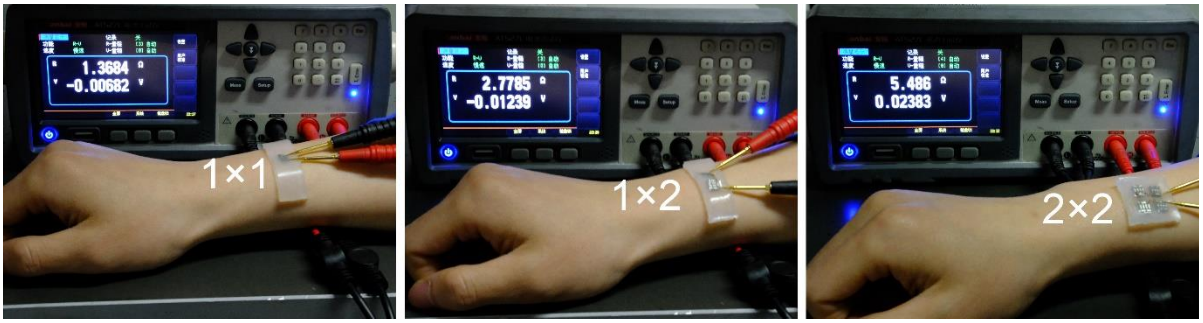

Figure S3 Photographs of the experimental setup for performance evaluation of the s-TEGs worn on a subject's wrist, including various TE Units arrays (i.e., 1×1, 1×2, and 2×2). The V_{OC} as well as the R_0 was measured using a commercial AC Electrical Load at steady-state conditions.

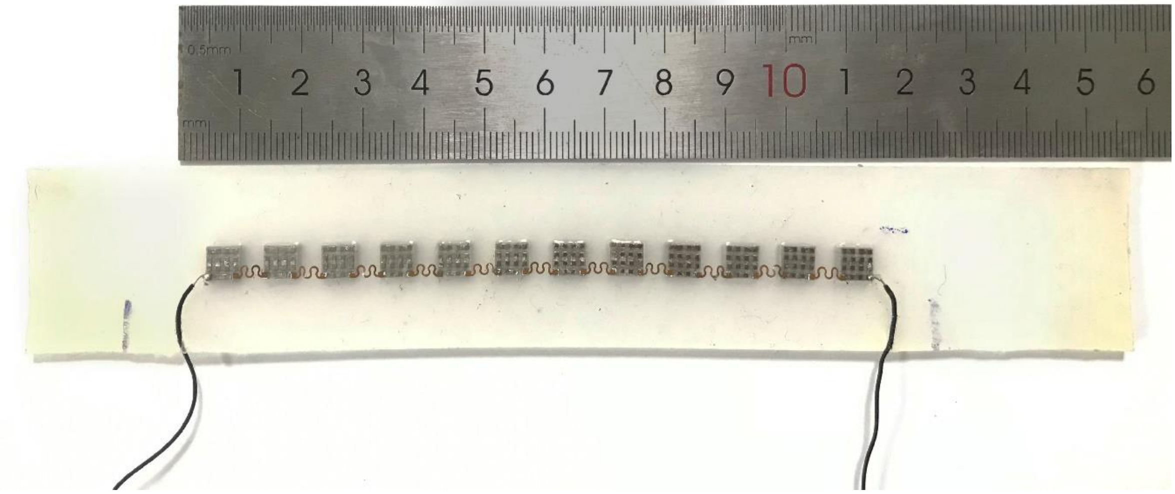

Figure S4 Photograph of the strip-shaped s-TEG with 12-TE Units connected in series in a row, which are distributed evenly in an area of about 11.5 cm × 0.6 cm.

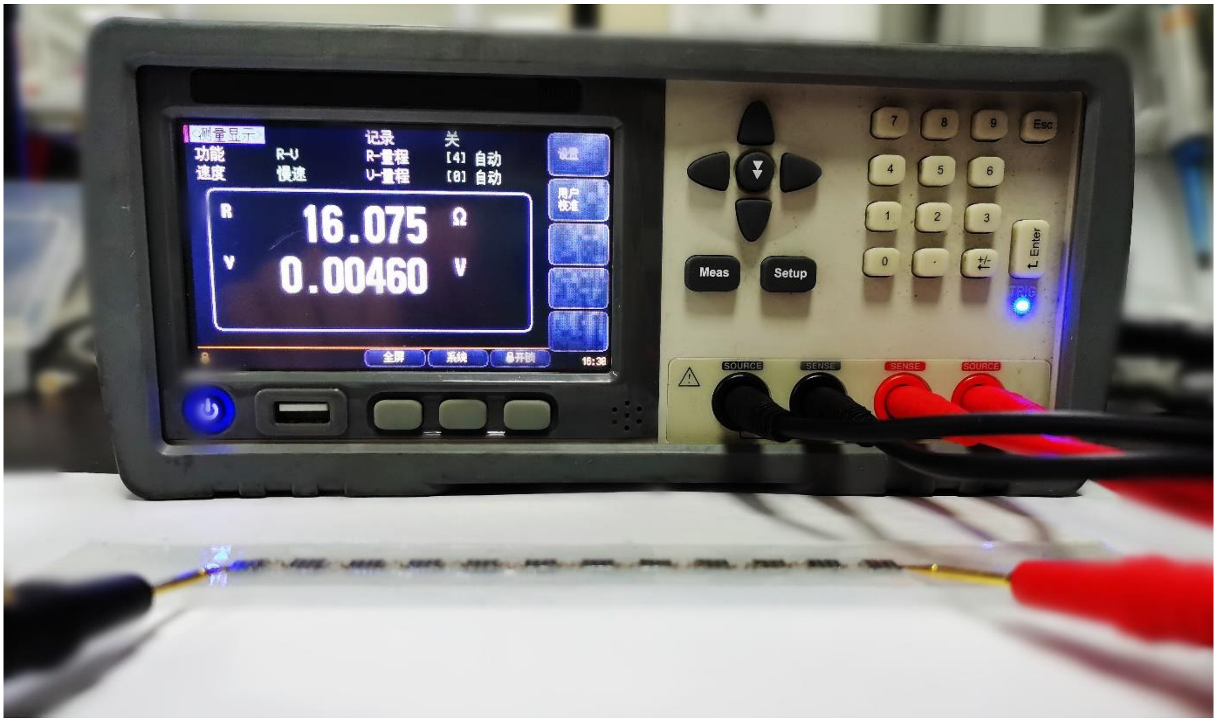

Figure S5 Photograph shows the measured R_0 of the s-TEG is about 16.08 Ω, which is in good accord with Fig. 4B.

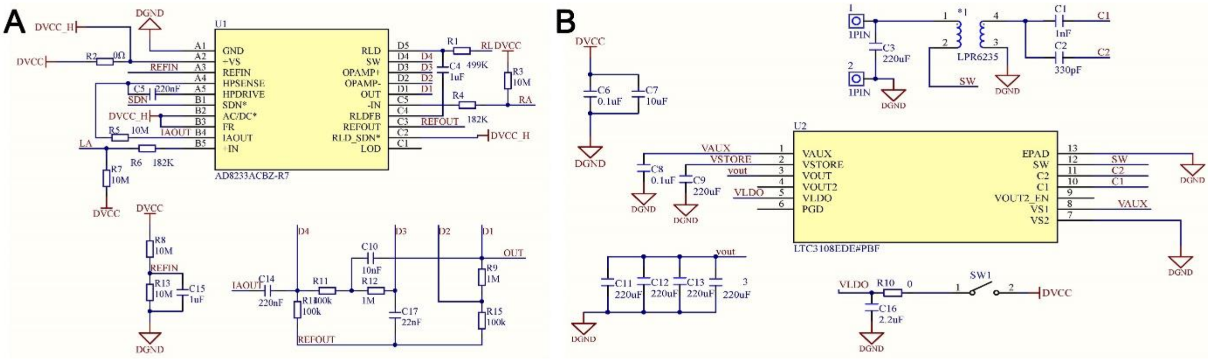

Figure S6 Detailed circuit diagrams of the ECG amplifier (A) and the boost converter (B).

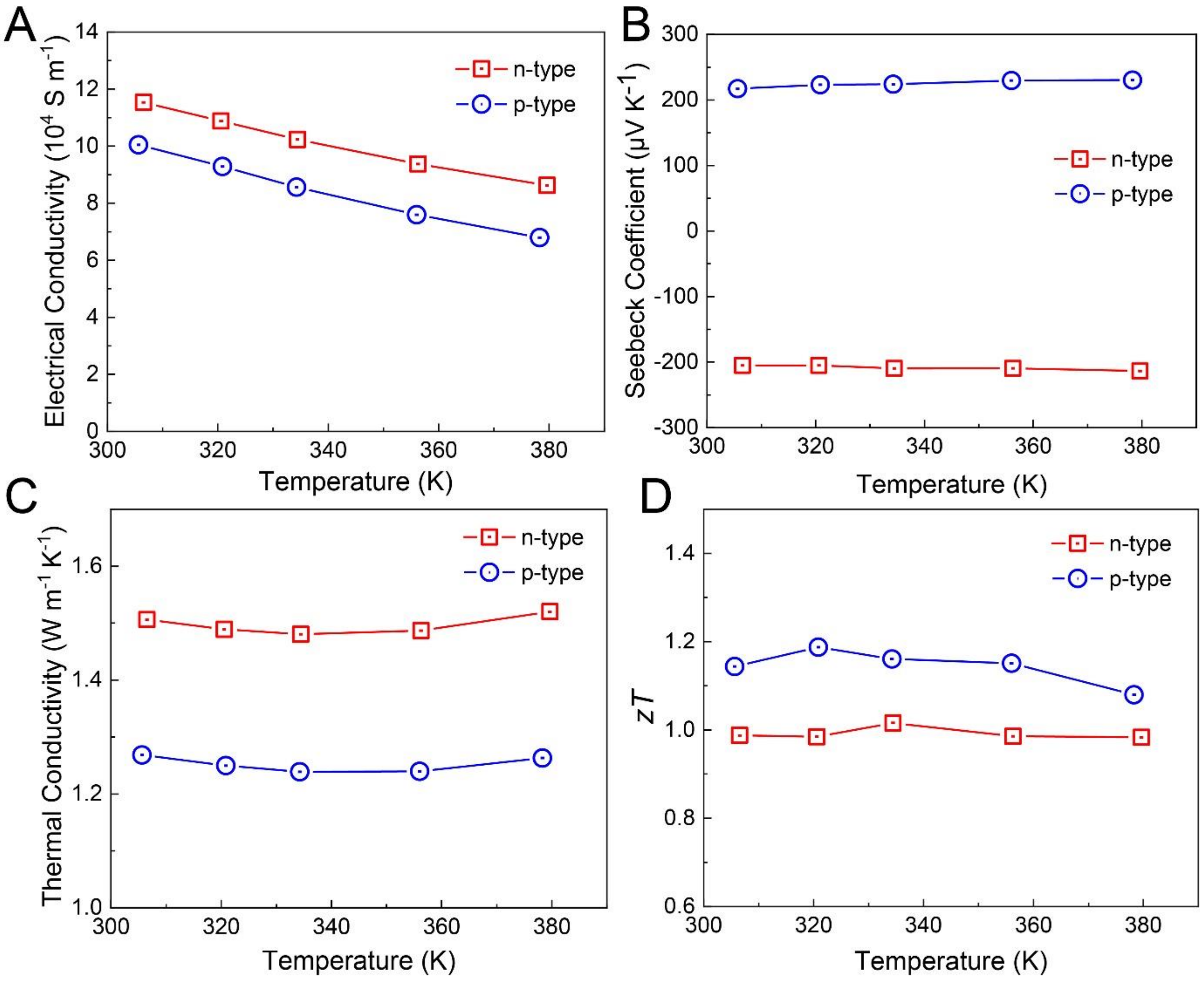

Figure S7 Measured thermoelectric properties of n- and p-type Bi_2Te_3 materials. (A) Electrical Conductivity. (B) Seebeck Coefficient. (C) Thermal Conductivity. (D) The figure of merit *zT*.

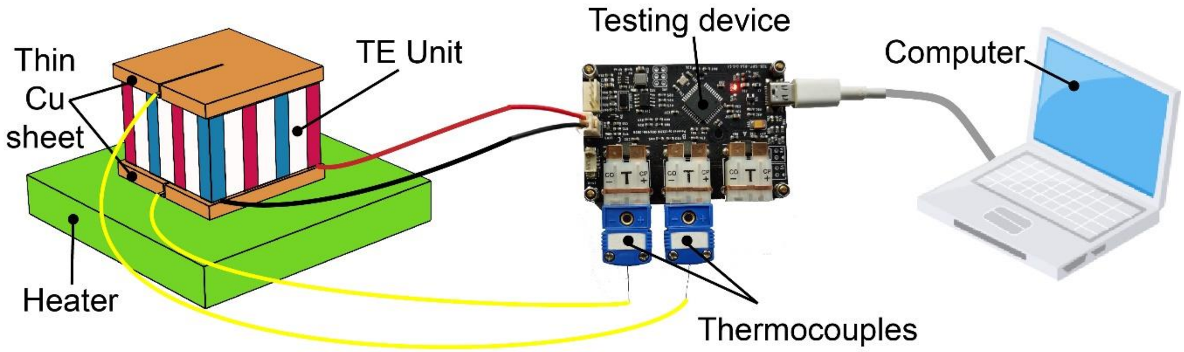

Figure S8 Schematic diagram of the homemade testing setup for output of TE Unit.

Table S1 Simulation parameters for finite element analysis (FEA).

**Material**	**Thermal conductivity(W m^{-1} K^{-1})**	**Height (μm)**
PDMS substrate*	0.16	120
Modified-PDMS substrate*	0.84	220
p-Bi_2Te_3	1.50	3000
n-Bi_2Te_3	1.27	3000
Ni	90.9	30
Au	318	1
Epoxy resin (filler)	0.05	3000

*The thermal conductivity and height of substrates, including PDMS substrate and Modified-PDMS substrate, were set according to previously reported studies.[S1-2]

Table S2 Comparison of device performance with previously reported TEGs with various substrates

Ref.	Substrate (thermal conductivity, W m^{-1} K^{-1})	Number of legs	Area (cm^2)	ΔT (K)	V_{oc} (mV)	Normalized Seebeck voltage per unit area (mV K^{-1} cm^{-2})	Normalized power density (μW cm^{-2} K^{-2})
This work	Substrate-free	16	0.3	30	84.9	9.45	5.13
S1	PDMS (0.16)	104	11.5	50	37.2	0.06	0.01
S3	Polyimide (typically < 0.3)	576	64	14	75	0.08	0.02
S4	silicone composite (0.28)	16	2.0	20	24	0.59	0.10
S5	Ag–Ni particles in PDMS (1.4)	440	16.8	40	2116	3.15	0.26
S6	Ecoflex/AlN (0.77)	144	25	8	172.1	0.86	0.59
S7	ceramic sheets (typically > 150)	254	16	64	2700	2.64	8.39

Table S3 Comparison of open circuit voltage and power density of wearable TEGs attached to human skin.

Ref.	Number of legs	Area (cm^2)	Ambient temperature and air speed	Open circuit voltage (mV)	Power density (μW cm^{-2})	Flexibility	Stretchability
This work	192	6.9	15 °C, breezeless condition	78.2	13.8		30%
			15 °C, V_{air} = 2 m s^{-1}	178.1	71.8		
S1	104	11.5	25 °C, breezeless condition	6.6	0.53	10 mm	
S5	440	16.8	NA	266	6.96		20 %
S6	144	25	22°C, breezeless condition	74.5	4.5	20 mm	
			10 °C, breezeless condition	165	10.7		
S8	64	4	24 °C, breezeless condition	1.47	0.37	5 mm	
			24 °C, V_{air} = 0.9 m s^{-1}	2.96	1.50		
S9	200	200	25 °C, V_{air} = 0.2 m s^{-1}	42	6.3	NA	
			15 °C, V_{air} = 2 m s^{-1}	138.5	48		

References

[S1] Y. Wang, Y. Shi, D. Mei, Z. Chen, *Appl. Energy* **2018**, 215, 690.

[S2] Y. Wang, Z. Zhou, J. Zhou, L. Shao, Y. Wang, Y. Deng, *Adv. Energy Mater.* **2021**, 12, 2102835.

[S3] J. Yuan, R. Zhu, *Appl. Energy* **2020**, 271, 115250.

[S4] S. H. Jeong, F. J. Cruz, S. Chen, L. Gravier, J. Liu, Z. Wu, K. Hjort, S. L. Zhang, Z. B. Zhang, *ACS Appl. Mater. Interfaces* **2017**, 9, 15791.

[S5] B. Lee, H. Cho, K. T. Park, J. S. Kim, M. Park, H. Kim, Y. Hong, S. Chung, *Nat. Commun.* **2020**, 11, 5948.

[S6] S. Hong, Y. Gu, J. K. Seo, J. Wang, P. Liu, Y. S. Meng, S. Xu, R. Chen, *Sci. Adv.* **2019**, 5, eaaw0536.

[S7] W. Zhu, P. Wei, J. Zhang, L. Li, W. Zhu, X. Nie, X. Sang, Q. Zhang, W. Zhao, *ACS Appl. Mater. Interfaces* **2022**, 14, 12276.

[S8] F. Suarez, D. P. Parekh, C. Ladd, D. Vashaee, M. D. Dickey, M. C. Öztürk, Appl. Energy **2017**, 202, 736.

[S9] Q. Xu, B. Deng, L. Zhang, S. Lin, Z. Han, Q. Zhou, J. Li, Y. Zhu, F. Jiang, Q. Li, P. Zhang, X. Zhang, G. Chen, W. Liu, *Cell Rep. Phys. Sci.* **2022**, 3, 100780.
